# Comprehensive developmental somatic proteome atlas of *Haemonchus contortus* underpinned by a chromosome-scale genome and deep tandem mass spectrometry

**DOI:** 10.1186/s13071-025-07050-0

**Published:** 2025-09-24

**Authors:** Tao Wang, Yuanting Zheng, Neil D. Young, Ching-Seng Ang, Robin B. Gasser

**Affiliations:** 1 Department of Veterinary Biosciences, Melbourne Veterinary School, Faculty of Science, The University of Melbourne, Parkville, VIC 3010 Australia; 2 Bio21 Mass Spectrometry and Proteomics Facility, The University of Melbourne, Parkville, VIC 3010 Australia

**Keywords:** *Haemonchus contortus*, Strongylida, Proteomics, Mass spectrometry, Parasitism, Developmental biology, Haemoglobin digestion, HIF-1 signalling

## Abstract

**Background:**

*Haemonchus contortus* is a highly pathogenic, blood-feeding nematode that causes widespread disease and significant economic loss in livestock worldwide. Previous proteomic studies were constrained by incomplete genomic resources and limited analytical sensitivity, impeding comprehensive profiling across life stages.

**Methods:**

In this study, we integrated advanced tandem mass spectrometry with a chromosome-scale genome assembly of the Haecon-5 strain to construct the most detailed somatic proteome of *H. contortus* to date.

**Results:**

We identified and quantified 7002 proteins across five key developmental stages/sexes—i.e. eggs, third-stage larvae (L3s), fourth-stage larvae (L4s), and adult female (Af) and adult male (Am) worms—tripling the number identified in an earlier study. Comparative analyses revealed pronounced stage-specific expression and functional specialisation, with parasitic stages enriched in proteins linked to metabolism, cellular function and environmental sensing. Fifteen proteins associated with the hypoxia-inducible factor 1 (HIF-1) signalling pathway were upregulated in parasitic stages, suggesting a role in adaptation to hypoxia. Additionally, 150 proteases implicated in haemoglobin degradation were characterised, and functional assays confirmed markedly elevated haemoglobinolytic activity in blood-feeding stages.

**Conclusions:**

These findings offer key insights into *H. contortus* development and parasitism, and establish a high-resolution proteomic framework to underpin fundamental biological studies and to enable the discovery of molecular targets for novel interventions against this and related nematodes.

**Graphical Abstract:**

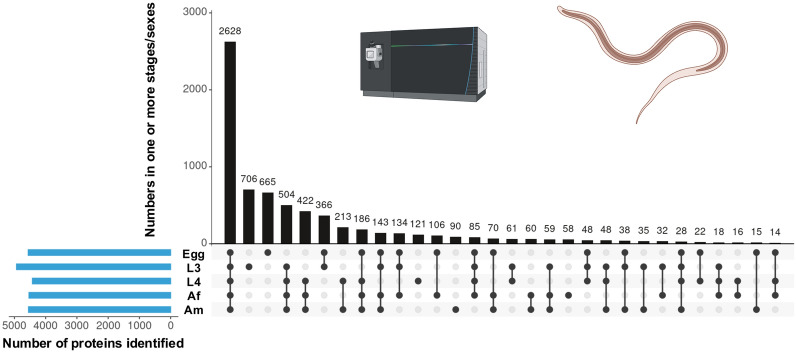

**Supplementary Information:**

The online version contains supplementary material available at 10.1186/s13071-025-07050-0.

## Background

Haematophagous parasitic nematodes cause significant disease in humans and animals, exerting a major impact on global health and agricultural productivity [1, 2]. For example, *Ancylostoma duodenale* and *Necator americanus* (hookworms) are blood-feeding soil-transmitted nematodes infecting approximately 472 million people worldwide, contributing to an estimated 4 million disability-adjusted life years (DALYs) [3]. In livestock, the barber’s pole worm—*Haemonchus contortus*—is among the most pathogenic parasites of small ruminants globally [1]. The control of *H. contortus* and related nematodes relies heavily on repeated use of relatively broad-spectrum anthelmintics. However, intensive drug administration has led to widespread resistance in multiple nematode species [4, 5]. There is an urgent need for new and sustainable interventions, informed by a deeper understanding of parasite development, host interaction and molecular biology.

Mass spectrometry-based proteomics, as a cornerstone of post-genomic biology, provides unique insight into protein abundance, temporal and cell-specific expression and post-translational modifications, features not readily accessible through genomics or transcriptomics alone [6]. Over the past decade, proteomic investigations have shed some light on parasite survival strategies, host immune evasion and immunomodulation. Most studies have focused on proteins at the host-parasite interface, including cuticular surface proteins, excretory/secretory proteins and extracellular vesicles [7–18]. In contrast, comparatively few studies have examined somatic proteomes across developmental stages of parasitic nematodes [19–21], leaving considerable gaps in our knowledge of their growth, reproduction and host adaptation.

Using high-throughput liquid chromatography-tandem mass spectrometry (LC-MS/MS) and a protein data set inferred from a nuclear genome assembled from short-read DNA data (BioProject: PRJEB2252; [22]), we previously characterised the first developmental somatic proteome of *H. contortus* [21]. However, the limited contiguity of the reference genome at that time [22] and genetic variability between strains (e.g. McMaster vs. Haecon-5) restricted protein identification to 2487 unique sequences—less than 25% of predicted proteins—across five key developmental stages and sexes—i.e. eggs; third- (L3s) and fourth-stage larvae (L4s); adult females (Af) and adult males (Am).

In the present study, we constructed a comprehensive developmental atlas of the somatic proteome of the Haecon-5 strain of *H. contortus* using deep tandem mass spectrometry, underpinned by a chromosome-scale genome of the same strain [23]. This atlas spans five critical stages and sexes of the parasite, providing unprecedented resolution of protein expression across the life cycle. By harnessing the chromosome-scale genome assembly [23] to underpin proteomic analysis, we resolved stage-specific protein expression and functional specialisation, including biological processes linked to parasitism such as blood feeding and environmental adaptation. This resource lays a solid foundation for fundamental, comparative and translational investigations and provides a valuable platform for the discovery of molecular targets in *H. contortus* and related parasitic nematodes.

## Methods

### Parasite material

*Haemonchus contortus* was produced in Merino lambs (6 months of age; Victoria, Australia), maintained under helminth-free conditions (animal ethics approval no. 23983; The University of Melbourne). Five different developmental stages/sexes of *H. contortus*—i.e. eggs, L3s, L4s, Af and Am—were produced. Briefly, sheep were inoculated via oral intubation with 10,000 infective L3s of *H. contortus*. One month after inoculation, eggs were isolated from freshly collected faeces by sucrose flotation at 22 °C within 4 h of collection [24]. Eggs were isolated from freshly collected faeces from infected sheep (1 month after inoculation) at 22^◦^ C within 4 h of collection using sucrose flotation [24]. L3s were cultured as described previously [25]. L4s and adult worms (Af and Am) were collected from the abomasa of infected sheep at 7 and 32 days after inoculation, respectively. Four biological replicates of each developmental stage were prepared, washed extensively (five times) in 50 ml volumes of physiological saline (pH 7.0), pelleted and frozen at –80 °C until use.

### Tissue homogenisation and protein extraction

Proteins were extracted from each of four replicates of each egg, L3, L4, Af and Am. In brief, approximately 30,000 eggs, 3000 larvae or 20 adults were transferred to Protein LoBind tubes (Eppendorf, Germany) containing 400 μl of lysis buffer (8 M urea in 100 mM triethylammonium bicarbonate, pH 8.5). Each sample was then subjected to sonication on ice at 20 kHz using a BioRuptor (eight cycles of 20 s on, 20 s off). Each sample was supplemented with protease inhibitor cocktail set I (Merck, Denmark) and incubated at 23 °C for 30 min. Samples were then centrifuged at 12,000 × *g* for 30 min at 4 °C, and the supernatants were collected for analysis. Protein concentrations were measured using a BCA Protein Assay Kit (Thermo Fisher Scientific, USA).

### In-solution digestion and LC-MS/MS analysis

To achieve maximum proteome coverage regarding the *H. contortus* genome, a hybrid data-independent acquisition (DIA) and data-dependent acquisition (DDA) approach was used as described previously to improve depth of coverage [21]. Individual samples from each stage (for DIA; containing 100 μg proteins) and pooled sample (for DDA-based spectrum libraries; containing 20 μg proteins of each egg, L3, L4, Af and Am) were reduced with 10 mM Tris (2-carboxyethyl) phosphine (TCEP) at 55 °C for 45 min and then alkylated with 55 mM iodoacetamide in the dark at 22 °C for 30 min, followed by a double enzymatic digestion with Lys-C/trypsin mix (Pierce, USA) at 37 °C for 16 h. The tryptic samples were acidified with 1.0% (v/v) formic acid, purified using Oasis HLB cartridges (Waters, USA). Proteins from the pooled sample were fractionated into eight parts using a high-pH reversed-phase peptide fractionation kit (Pierce, USA), according to the manufacturer’s protocol for spectra library generation. All digested peptide samples were freeze-dried and re-suspended in aqueous 2% w/v acetonitrile and 0.05% trifluoroacetic acid (TFA) w/v before LC-MS/MS analysis.

### Data-dependent LC-MS/MS

LC-MS/MS was carried out using a Orbitrap Ascend mass spectrometer (Thermo Scientific) equipped with nanoflow reversed-phase-HPLC (Ultimate 3000 RSLC, Dionex) fitted with an Acclaim Pepmap nano-trap column (Dionex—C18, 100 Å, 75 µm × 2 cm) and an Acclaim Pepmap RSLC analytical column (Dionex-C18, 100 Å, 75 µm × 50 cm). The tryptic peptides were injected to the enrichment column at an isocratic flow of 5 µl/min of 2% v/v CH_3_CN containing 0.1% v/v formic acid for 5 min, after which the enrichment column was switched in line with the analytical column. The eluents were 5% DMSO in 0.1% v/v formic acid (solvent A) and 5% DMSO in 100% v/v CH_3_CN and 0.1% v/v formic acid (solvent B). The flow gradient was (i) 0–6 min at 3% B; (ii) 6–7 min, 3–4%; (ii) 7–82 min, 4–25% B; (iii) 82–86 min 25–40% B; (iv) 86–87 min, 40–80% B; (v) 87–90 min, 80–80% B; and (vi) 90–91 min, 80–3%, equilibrated at 3% B for 10 min before the next sample injection. The mass spectrometer was operated in data-dependent acquisition mode, whereby complete MS1 spectra were acquired in positive mode at a resolution of 120,000. The ‘top speed’ acquisition mode (3 s cycle time) on the most intense precursor ion was used (i.e. charge states of 2 to 7). MS/MS analyses were performed by 1.6 m*/z* isolation with the quadrupole, fragmented by HCD with normalized collision energy of 30%. MS2 fragmented ion spectra were acquired at a resolution of 15,000. Dynamic exclusion was activated for 30 s, and AGC target was set to standard with auto maximum injection mode.

### Data-independent LC-MS/MS

For DIA experiments, full MS resolutions were set to 120,000 at m/z 200, with scanning performed from 350–1400 m/z in profile mode. The full MS AGC target was set to 250% with an IT of 50 ms. The AGC target value for fragment spectra was set to 2000%. Fifty windows of 13.7 Da were used with an overlap of 1 Da. Resolution was set to 30,000 and maximum IT to 55 ms. Normalised collision energy was set to 30%. All data were acquired in centroid mode using positive polarity.

### Protein identification and quantification

DIA data were analysed using the hybrid direct DIA analysis in combination of workflow with default settings on the Spectronaut software (v.19.4.241104.62635) and against the proteome predicted from the full-length chromosome-contiguous genome of *H. contortus* of the Haecon-5 strain [23]. Trypsin specificity was set to allow up to two missed cleavages. Carbamidomethylation of cysteine was defined as a fixed modification, and N-terminal acetylation and methionine oxidation were set as variable modifications. Results were filtered at a Protein and PSM false discovery rate (FDR) of 1%. Precursor filtering using the q-values and quantification was carried out on the MS2 level, and the cross-run normalisation strategy was set to automatic. Quantitative data were exported for statistical analyses using Perseus (v.1.6.1.1) [26]. Only proteins identified in ≥ 3 biological replicates of at least one developmental stage were accepted. The mass spectrometry proteomic data (identifier PXD066296) are accessible via the PRIDE archive of the ProteomeXchange Consortium [27].

### Fluorescamine derivatisation assay to assess the proteolysis of haemoglobin

The ability of peptidases in protein extracts from individual developmental stages/sexes of *H. contortus* to lyse haemoglobin was evaluated. Aliquots (1 µg) of individual extracts were incubated (in triplicate) with 20 µg of bovine haemoglobin (Sigma, USA) in 100 mM sodium acetate, 25 mM DTT and 25 mM NaCl, in a total volume of 100 µl (pH 4.5) at 37°C for 16 h. After incubation, 50 µl of fluorescamine solution (0.03% in acetone) was added to assess newly formed N-terminal amino groups [28]. The fluorescence signal in each sample was measured using a Synergy H1 plate reader at 370 nm excitation and 485 nm emission wavelengths. Each analysis was performed in triplicate.

### Bioinformatic analyses

The UniProt repository was used for protein annotation, including cellular compartment, subcellular location, transmembrane region and molecular function. Molecular functions of proteins quantified in the proteome of *H. contortus* were classified according to Gene Ontology (GO) using InterProScan [29]. UpSet plot was drawn using Intervene [30]. Principal component analysis (PCA) and hierarchical cluster analysis (HCA) were performed using Perseus software (v.1.6.1.1) using default settings [26]. Biological functions were assigned to differentially expressed proteins using Eggnog-mapper v.2.1.9 [31]. Pathway annotation was analysed using KO terms from Kyoto Encyclopedia of Genes and Genomes (KEGG) database [32]. Enriched KEGG pathways were identified using TBtools [33] with a cut-off of adjusted *p*-value ≤ 0.05. InterProScan was also used to verify KEGG annotations and resolve potential errors [34]. The enrichment results were displayed in dot plot based on a custom-modified Enrichplot package in R script. Statistical comparisons across groups were performed using one-way ANOVA in GraphPad Prism 10.4.1 (GraphPad, La Jolla, CA, USA), with statistical significance defined as *p* < 0.05.

## Results

### Comprehensive somatic proteome atlas of *H. contortus*

**Fig. 1:**
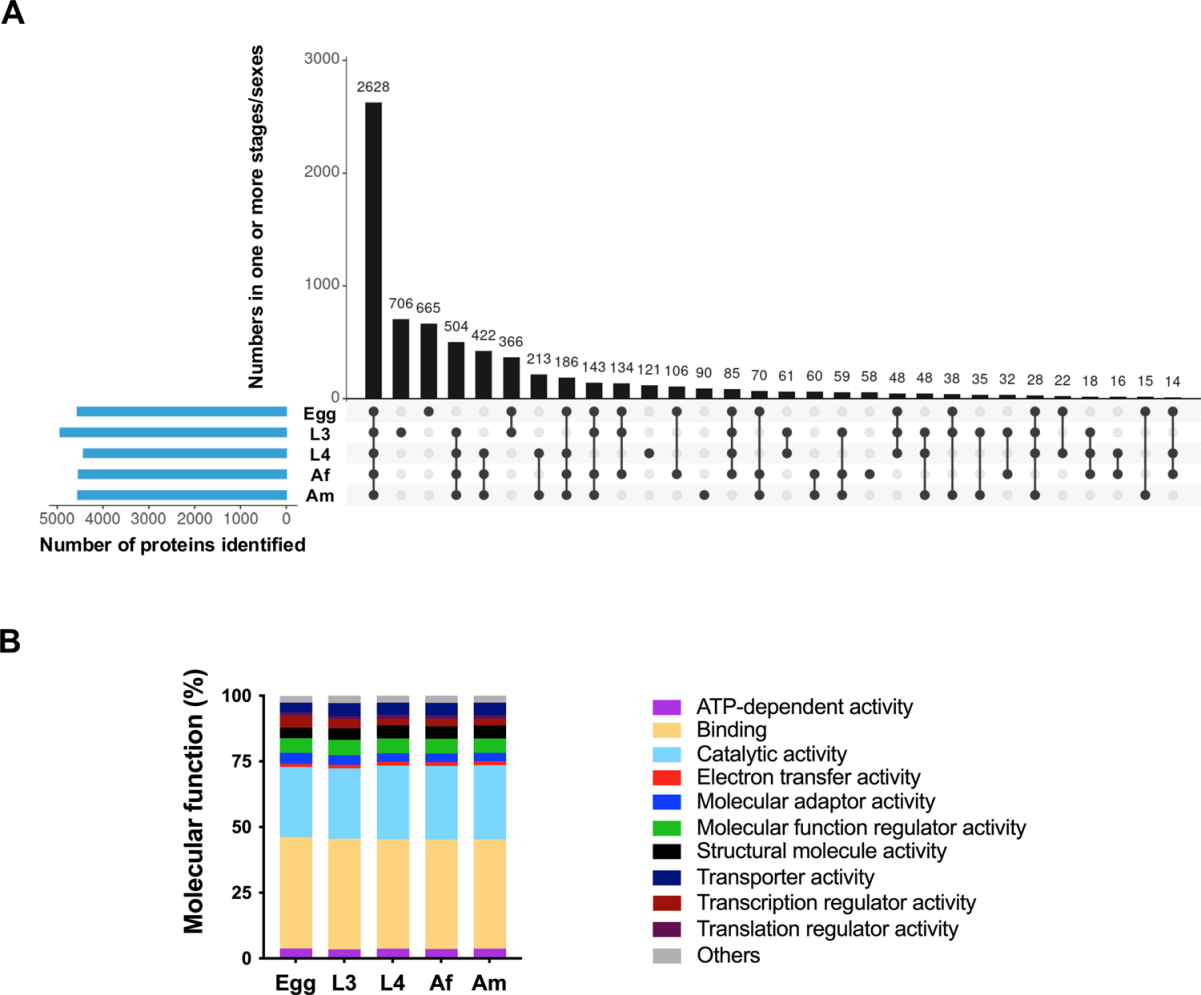
(**A**) UpSet plot showing the number of somatic proteins unique to, or shared among, five key developmental stages/sexes of *Haemonchus contortus*: egg, third-stage larva (L3), fourth-stage larva (L4), adult female (Af) and adult male (Am). Vertical bars represent shared or unique protein counts across one or more stages/sexes, as indicated by the connected dots below. Horizontal bars indicate the total number of proteins identified in each stage or sex. **B** Functional classification (Gene Ontology, GO – level 2) of identified proteins by molecular function. Values are expressed as the percentage of total proteins detected in each stage or sex, allowing direct comparisons of the distribution of functional categories among the five developmental stages/sexes (see Table S2)

A total of 7002 somatic proteins were identified and quantified across five key developmental stages and sexes of *H. contortus* (i.e. egg, L3, L4, Af and Am). The distribution of proteins across these stages is summarised in Fig. 1A, and the complete list is given in Table S1. The largest numbers of proteins were detected in the egg (*n* = 5661) and L3 (*n* = 5567) stages, followed by Af (*n* = 5019), Am (*n* = 4795) and L4 (*n* = 4728). Most proteins (*n* = 5362; 77%) were shared between at least two stages, with 2628 proteins (38%) expressed across all five stages and sexes. Stage-specific protein sets were also observed. L3 and egg had the highest numbers of unique proteins (*n* = 706 and *n* = 665, respectively), whereas 121, 90 and 58 proteins were uniquely detected in L4, Am and Af, respectively (Fig. 1A).

### Functional classification of somatic proteins across developmental stages

The distribution of molecular functions (Gene Ontology [GO] – level 2) for proteins identified in each developmental stage and sex of *H. contortus* is summarised in Fig. 1B. Annotation revealed that most proteins were associated with binding (GO:0005488; 41.7–42.5%) and catalytic activity (GO:0003824; 26.7–28.1%) across all stages. Each of these two functional categories comprised > 1300 annotated proteins per stage.

Other GO molecular function classes—including ATP-dependent activity, molecular adaptor activity, structural molecule activity and transporter activity—were consistently less represented. Within the binding category, major subclasses included compound binding (e.g. sulphur and organic cyclic compounds), protein binding, small molecule binding and carbohydrate derivative binding. Proteins classified under catalytic activity were predominantly associated with transferase and hydrolase functions (Table S2). The relative distribution of molecular function categories was broadly conserved across developmental stages and sexes, with no major shifts observed (Fig. 1B).

### Differential protein expression across developmental stages and sexes

**Fig. 2:**
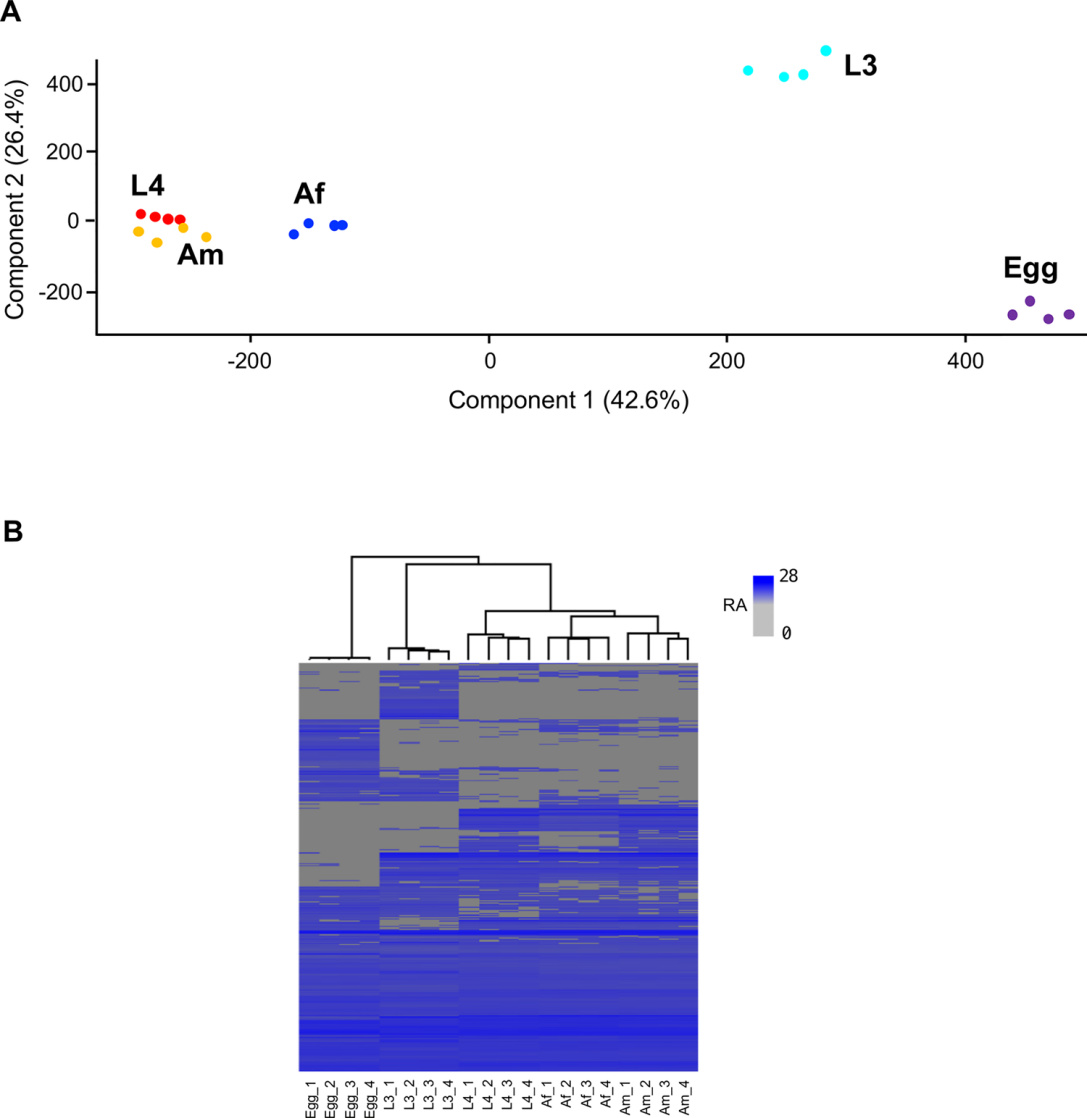
**A** Principal component analysis (PCA) of somatic protein abundance across five developmental stages or sexes of *Haemonchus contortus*: egg, third-stage larva (L3), fourth-stage larva (L4), adult female (Af) and adult male (Am). Each point represents a biological replicate, highlighting consistency within stages or sexes and differences between them. **B** Heatmap showing hierarchical clustering of stage- and sex-specific expression profiles based on normalised protein abundance. The grey-to-blue colour scale indicates low-to-high abundance. Each stage or sex is represented by four biological replicates. RA, relative abundance

To gain further insight into the somatic proteome of *H. contortus*, we performed a principal component analysis (PCA) based on protein abundance profiles across five developmental stages and sexes (i.e. egg, L3, L4, Af and Am; Fig. 2A). The two-dimensional PCA revealed tight clustering of biological replicates within each group, indicating strong within-stage consistency.

The three parasitic stages (L4, Af and Am) clustered closely together, to the exclusion of the free-living stages (egg and L3). Notably, the proteomic divergence between egg and L3 was more pronounced than that among the parasitic stages, suggesting substantial molecular transitions even within the pre-parasitic phase of development. Consistent with PCA, hierarchical clustering of normalised protein abundance (Fig. 2B) confirmed greater similarity among the parasitic stages than between parasitic and free-living stages. Together, these analyses demonstrate extensive alterations in the somatic proteome of *H. contortus* during development and sexual differentiation.

**Fig. 3:**
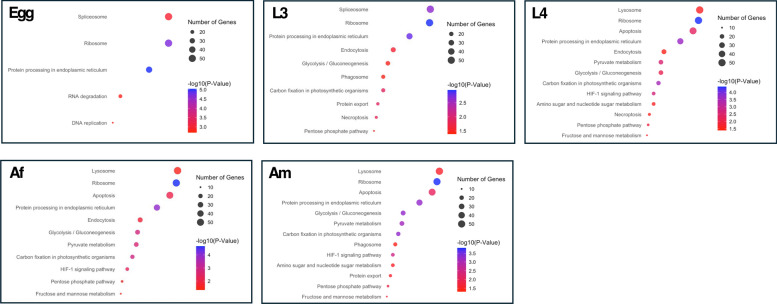
Dot plots showing enriched KEGG biological pathways associated with differentially expressed proteins across five developmental stages and sexes of *Haemonchus contortus*—egg, third-stage larva (L3), fourth-stage larva (L4), adult female (Af) and adult male (Am). Each panel displays stage-specific pathway enrichment, with dot size representing the number of proteins assigned to a given pathway and dot colour indicating statistical significance (− log₁₀ adjusted *p*-value). The x-axis shows the protein ratio, defined as the proportion of proteins in each pathway relative to the total number of proteins identified for that stage. Only pathways with an adjusted *p*-value ≤ 0.05 were considered significantly enriched (see Supplementary Table S3 for detailed results)

KEGG pathway annotation inferred that proteins differentially expressed among the five developmental stages/sexes were classified into biological categories: environmental information processing, cellular processes, metabolism and genetic information processing (Fig. 3; Table S3). In the egg stage, a large proportion of abundantly expressed proteins associated with genetic information processing (*n* = 139), predominantly assigned to translation (52; ribosome), transcription (52; spliceosome) and folding, sorting and degradation (20; RNA degradation). In contrast, the larval and adult stages exhibited stage-specific features. Proteins enriched in the L3 stage were linked predominantly to genetic information processing (*n* = 154), representing transcription (52; spliceosome), translation (51; ribosome) and folding, sorting and degradation (51; protein processing and protein export), followed by cellular processes (56) and metabolism (50).

In the L4 stage, most enriched proteins were associated with cellular processes (*n* = 132), particularly transport and catabolism (74) and cell growth and death (58), as well as metabolism (97), which included carbohydrate metabolism (64), energy metabolism (19) and glycan biosynthesis and metabolism (15). In adult stages, proteins enriched in Af were primarily associated with cellular processes (127), linked exclusively to transport and catabolism (79; lysosome and endocytosis) and cell growth and death (48; apoptosis). Proteins enriched in Am were assigned to both cellular processes (115) and metabolism (100). Within the cellular process category, Am-enriched proteins were involved predominantly in transport and catabolism (69; lysosome and phagosome) and cell growth and death (46; apoptosis). In metabolism, carbohydrate metabolism (66) was dominant, followed by energy (19) and glycan (15) metabolism. Notably, a subset of enriched somatic proteins was associated with environmental information processing, specifically the HIF-1 signalling pathway (*n* = 15), in the three parasitic stages (L4, Af and Am).

### Assessing the haemoglobinolytic capacity of somatic proteins

**Fig. 4:**
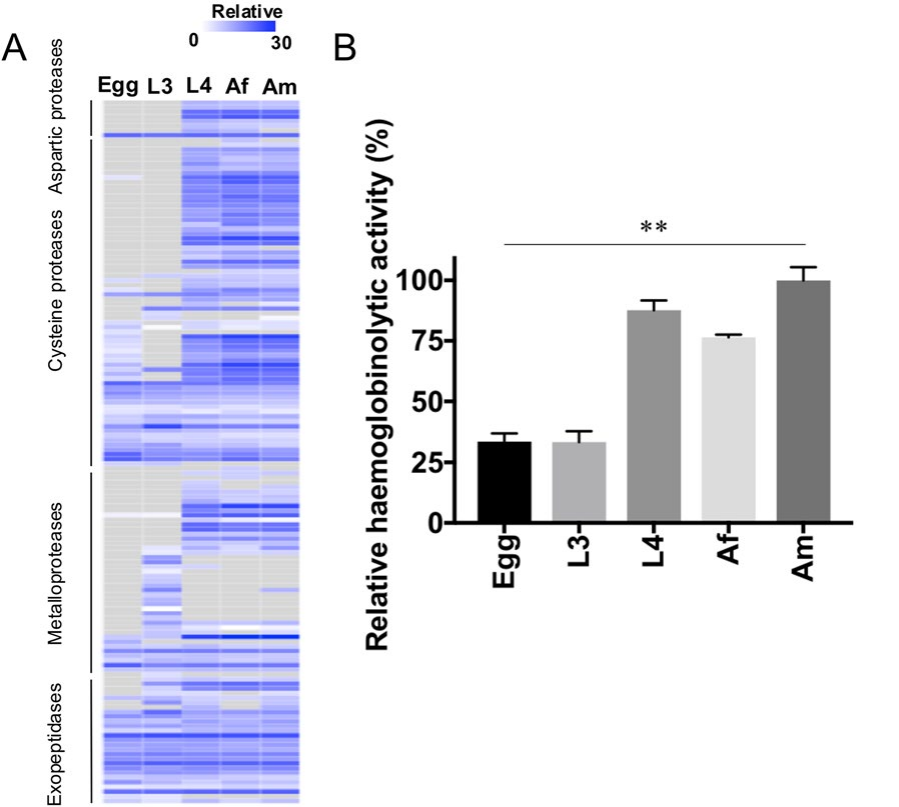
**A** Heatmap of putative haemoglobin-degrading proteases identified in the somatic proteome of *Haemonchus contortus* across five developmental stages and sexes—egg, third-stage larva (L3), fourth-stage larva (L4), adult female (Af) and adult male (Am). Proteases are grouped by class (aspartic, cysteine, metallopeptidase and exopeptidase), and normalised protein abundance is shown on a grey-to-blue scale representing low-to-high abundance. **B** Relative haemoglobinolytic activity of somatic protein extracts, quantified by fluorescamine derivatisation. Activity levels are expressed as a percentage relative to the maximum value (Am = 100%), with bars representing the mean ± standard deviation (SD) for each stage. Proteolytic activity was significantly higher in the blood-feeding stages (L4, Af and Am) compared with the non-blood-feeding stages (egg and L3) (*p* < 0.01, ANOVA)

Building on prior observations of protease enrichment in parasitic stages of *H. contortus*, we examined the developmental distribution and activity of enzymes likely involved in haemoglobin degradation. Across all five developmental stages and sexes of *H. contortus*, we identified 150 candidate proteases, including aspartic proteases (*n* = 8), cysteine proteases (70), metallopeptidases (44) and exopeptidases (28). These proteases, particularly cysteine proteases and metallopeptidases, showed markedly higher abundance in the parasitic stages—L4, Af and Am—compared with the non-parasitic egg and L3 stages (Fig. 4A), coinciding with the onset of blood feeding.

To assess whether this pattern translated into functional activity, we performed a fluorescamine derivatisation assay to measure haemoglobinolytic capacity in somatic extracts from each stage. The results revealed a strong developmental trend: activity was highest in adult males (Am; 100%), followed by L4 (87.7%) and adult females (Af; 76.5%), and substantially lower in the non-blood-feeding stages, egg (33.6%) and L3 (33.4%) (Fig. 4B). Statistical analysis confirmed that proteolytic activity was significantly elevated in parasitic stages (*p* < 0.01, ANOVA). Taken together, these findings demonstrate that the transition to parasitism in *H. contortus* involves both proteomic enrichment and functional activation of haemoglobin-degrading enzymes. This developmental upregulation reflects the parasite’s adaptation to a blood-feeding lifestyle and identifies key protease families with potential relevance as therapeutic targets.

## Discussion

This study presents the most comprehensive and stage-resolved somatic proteome of *H. contortus* reported to date, offering a high-resolution view of protein expression profiles across the parasite’s life cycle. Leveraging a chromosome-scale genome assembly [23] and deep mass spectrometry, this atlas quantifies 7002 proteins—representing a threefold increase over an earlier study [21]. It provides a valuable framework for exploring the molecular mechanisms underpinning parasite development, adaptation and survival and lays the foundation for both basic and applied research.

A particularly notable finding was the pronounced divergence in somatic protein expression between free-living (egg and L3) and parasitic (L4, adult female and adult male) stages of *H. contortus*. These transitions reflect the organism’s shift from environmental persistence/survival to blood feeding, immune evasion and reproduction. Principal component and hierarchical clustering analyses revealed that the proteomes of parasitic stages converge around a shared functional core, and the free-living stages exhibit distinct expression signatures—most notably, the divergence between egg and L3 stages, suggesting developmental reprogramming well before host entry.

Functionally, these shifts were reflected in KEGG pathway enrichments. The egg and L3 stages were dominated by proteins associated with genetic information processing, supporting high levels of transcriptional and translational activity. In contrast, L4 and adult stages showed broad activation of cellular regulation, metabolism and environmental sensing, particularly pathways or processes linked to nutrient acquisition and host adaptation. Of these, the hypoxia-inducible factor 1 (HIF-1) signalling pathway stood out, with 15 associated proteins enriched specifically in parasitic stages. This observation resonates with prior work in *Caenorhabditis elegans*, where HIF-1 mediates cellular adaptation to low oxygen via the EGL-9/VHL-1/RHY-1 module [35]. Interestingly, in *Ascaris suum*, *hif-1* expression peaks in the L3 stage (from the egg) [36], suggesting a “preparatory” mechanism for transition into the host animal. In contrast, our results indicate that in *H. contortus*, HIF-1 pathway proteins are upregulated after host entry, pointing to species-specific regulatory strategies for adaptation to hypoxia. This provides an interesting avenue for functional dissection of oxygen-sensing networks in parasitic nematodes.

Another defining biological feature of *H. contortus* is its blood-feeding behaviour in the host animal, which is at the L4 stage. As in other haematophagous parasitic nematodes (e.g. species of *Ancylostoma* and *Necator*), proteases are regarded as essential enzymes for haemoglobin degradation and anticoagulation in *H. contortus* [37, 38]. We identified 150 putative haemoglobin-degrading proteases spanning aspartic, cysteine, metallopeptidase and exopeptidase classes. Consistent with a previous study [21], these proteases were abundantly expressed in L4, Af and Am stages, coinciding with the onset and progression of blood ingestion. Importantly, this expression profile overall was matched by function in that fluorescamine derivatisation assays revealed significantly higher haemoglobinolytic activity in the parasitic stages compared with egg and L3 stages. These findings provide direct evidence that stage-specific protease expression translates into functional capacity for haemoglobin degradation. Together, these results reinforce the role of coordinated protease activity as a core molecular feature of parasitism by *H. contortus.*

Proteases involved in blood digestion are already recognised as prime targets for intervention. Native gut-derived protease complexes, such as H11 and H-gal-GP, have demonstrated efficacy in experimental vaccine trials against haemonchosis [39, 40], and recombinant aspartic protease-1 has been under clinical evaluation as part of a human hookworm vaccine (e.g. [41]). Our findings expand the catalogue of functionally validated candidates and provide detailed, stage-resolved expression profiles across multiple protease families. The presence of multiple, co-expressed protease classes suggests potential redundancy and synergistic function, enhancing parasite resilience. Understanding how these enzymes are regulated and functionally integrated during parasitism could reveal molecular targets amenable to therapeutic intervention.

Beyond these stage-specific processes, this study also raises broader questions regarding nematode evolution and functional adaptation. The differential activation of core pathways, such as HIF-1 signalling, lysosomal processing and carbohydrate metabolism, suggests modular reprogramming of the somatic proteome in response to host-derived cues. These patterns may be conserved across other blood-feeding nematodes, such as *Ancylostoma* and *Necator*, or may reflect specific features in *H. contortus*. Comparative proteomic studies across nematode clades, enabled by this atlas, could resolve such questions and provide evolutionary context for parasitic traits.

## Conclusions

This comprehensive, stage-resolved proteomic atlas of *H. contortus*, underpinned by a high-quality chromosome-scale genome, captures the molecular architecture of developmental and parasitic transitions. It highlights hypoxia response and haemoglobin digestion as hallmarks of parasitism and identifies protease families of clear translational relevance. With thousands of proteins annotated by developmental stage and predicted function, this resource provides a high-resolution framework for functional investigations. Gene-to-phenotype relationships could now be explored using RNA interference, CRISPR-based editing, proximity labelling and spatial proteomics. These investigations will be instrumental in defining the roles of parasite-specific and stage-enriched proteins in development, metabolism, immune modulation and host interaction. Beyond advancing fundamental understanding, this atlas opens new avenues for the discovery and validation of candidate targets for vaccines and therapeutics against *H. contortus* and other haematophagous nematodes.

## Supplementary Information

Additional file 1. Table S1. Complete list of proteins quantified in the *Haemonchus contortus* proteome of five different developmental stages/sexes (i.e. egg, third-stage larva [L3], fourth-stage larva [L4], adult female [Af] and adult male [Am])

Additional file 2. Table S2. Numbers of quantified proteins involved in the molecular function (levels 2 and 3 – Gene Ontology (GO) categories) in five different developmental stages/sexes of *Haemonchus contortus*—i.e. egg, third-stage larva (L3), fourth-stage larva (L4), adult female (Af) and adult male (Am).

Additional file 3. Table S3. Enriched KEGG pathways of differentially expressed proteins in five different developmental stages/sexes of *Haemonchus contortus*—i.e. egg, third-stage larva (L3), fourth-stage larva (L4), adult female (Af) and adult male (Am).

## Data Availability

Data supporting the conclusions of this article are included within the article.

## Abbreviations

Af: Adult female worms
Am: Adult male worms
DALYs: Disability-adjusted life years
DIA: Data-independent acquisition
DDA: Data-dependent acquisition
FDR: False discovery rate
GO: Gene ontology
HCA: Hierarchical cluster analysis
L3: Third-stage larva
L3s: Third-stage larvae
L4: Fourth-stage larva
L4s: Fourth-stage larvae
PCA: Principal component analysis
KEGG: Kyoto Encyclopedia of Genes and Genomes
SD: Standard deviation
TCEP: Tris (2-carboxyethyl) phosphine
TFA: Trifluoroacetic acid
